# Correlations between serum laminin level and severity of heart failure in patients with chronic heart failure

**DOI:** 10.3389/fcvm.2023.1089304

**Published:** 2023-03-16

**Authors:** Ling Xie, Zhen Zhou, Hai-Xiao Chen, Xiao-Yun Yan, Jia-Qi Ye, Ying Jiang, Lei Zhou, Qing Zhang

**Affiliations:** ^1^ Department of Cardiology, Second Affiliated Hospital of Nantong University, Nantong, China; ^2^ Deparment of Science and Education, Nantong Third People’s Hospital, Nantong, China; ^3^ Department of General Medicine, Second Affiliated Hospital of Nantong University, Nantong, China; ^4^ Department of Cardiology, First Affiliated Hospital of Nanjing Medical University, Nanjing, China

**Keywords:** serum laminin, chronic heart failure, degree of severity, stages of heart failure, correlations

## Abstract

**Objective:**

This study aimed to investigate the correlation between serum laminin (LN) levels and clinical stages of heart failure in patients with chronic heart failure.

**Methods:**

A total of 277 patients with chronic heart failure were selected from September 2019 to June 2020 in the Department of Cardiology, Second Affiliated Hospital of Nantong University. Based on stages of heart failure, the patients were divided into four groups: stage A, stage B, stage C, and stage D, with 55, 54, 77, and 91 cases, respectively. At the same time, 70 healthy people in this period were selected as the control group. Baseline data were recorded and serum Laminin (LN) levels were measured. The research compared, the differences in baseline data among the four groups of HF and normal controls, and analyzed the correlation between N-terminal pro-brain natriuretic peptide (NT-proBNP) and left ventricular ejection fraction (LVEF). The receiver operating characteristic (ROC) curve was used to evaluate the predictive value of LN in the C-D stage of heart failure. Logistic multivariate ordered analysis was applied to screen the independent related factors of clinical stages of heart failure.

**Results:**

Serum LN levels in patients with chronic heart failure were significantly higher than those in healthy people, which were 33.2 (21.38, 101.9) ng/ml and 20.45 (15.53, 23.04) ng/ml, respectively. With the progression of clinical stages of HF, serum LN and NT-proBNP levels increased, while LVEF gradually decreased (*P *< 0.05). Correlation analysis showed that LN was positively correlated with NT-proBNP (*r* = 0.744, *P *= 0.000) and negatively correlated with LVEF (*r* = −0.568, *P *= 0.000). The area under the ROC curve of LN for predicting C and D stages of heart failure was 0.913, 95% confidence interval was 0.882–0.945, *P *= 0.000, specificity 94.97%, and sensitivity 77.38%. Multivariate Logistic analysis showed that LN, Total bilirubin, NT-proBNP and HA were all independent correlates of heart failure staging.

**Conclusion:**

Serum LN levels in patients with chronic heart failure are significantly increased and are independently correlated with the clinical stages of heart failure. It could potentially be an early warning index of the progression and severity of heart failure.

## 1. Introduction

Chronic heart failure (CHF) is one of the most common chronic diseases worldwide, and it has seen an increasing trend in its prevalence and morbidity (1). Ventricular remodeling is an important pathological basis of CHF, and inhibition of its process is beneficial to improve the prognosis of patients with heart failure (HF) (2). Myocardial fibrosis is one of the primary manifestations of ventricular remodeling. It has been found that myocardial fibrosis mainly features changes in the extracellular matrix (ECM) (3). Laminin (LN) is a vital component of ECM, which has significant biological functions, including adhesion, translocation, cell differentiation, cell growth, and inflammatory response (4). Many studies have shown that LN is closely related to the process of myocardial fibrosis (5–7). The 2022 AHA/ACC/HFSA guidelines (8) divide HF into four stages (stages A–D) according to HF progression, which objectively reflects the clinical progress of CHF. Early risk factor control and active drug treatment can prevent and delay HF progression to the greatest extent. Therefore, the identification of markers related to HF progression is of particular significance. To date, on study has focused on the correlation between LN and the clinical stage of CHF. This study aimed to explore the correlation between serum LN and HF severity of heart failure in patients with CHF by analyzing serum LN levels in patients with different stages of CHF.

## 2. Manuscript formatting

### 2.1. Methods

#### 2.1.1. Study design and participants

**Figure 1:**
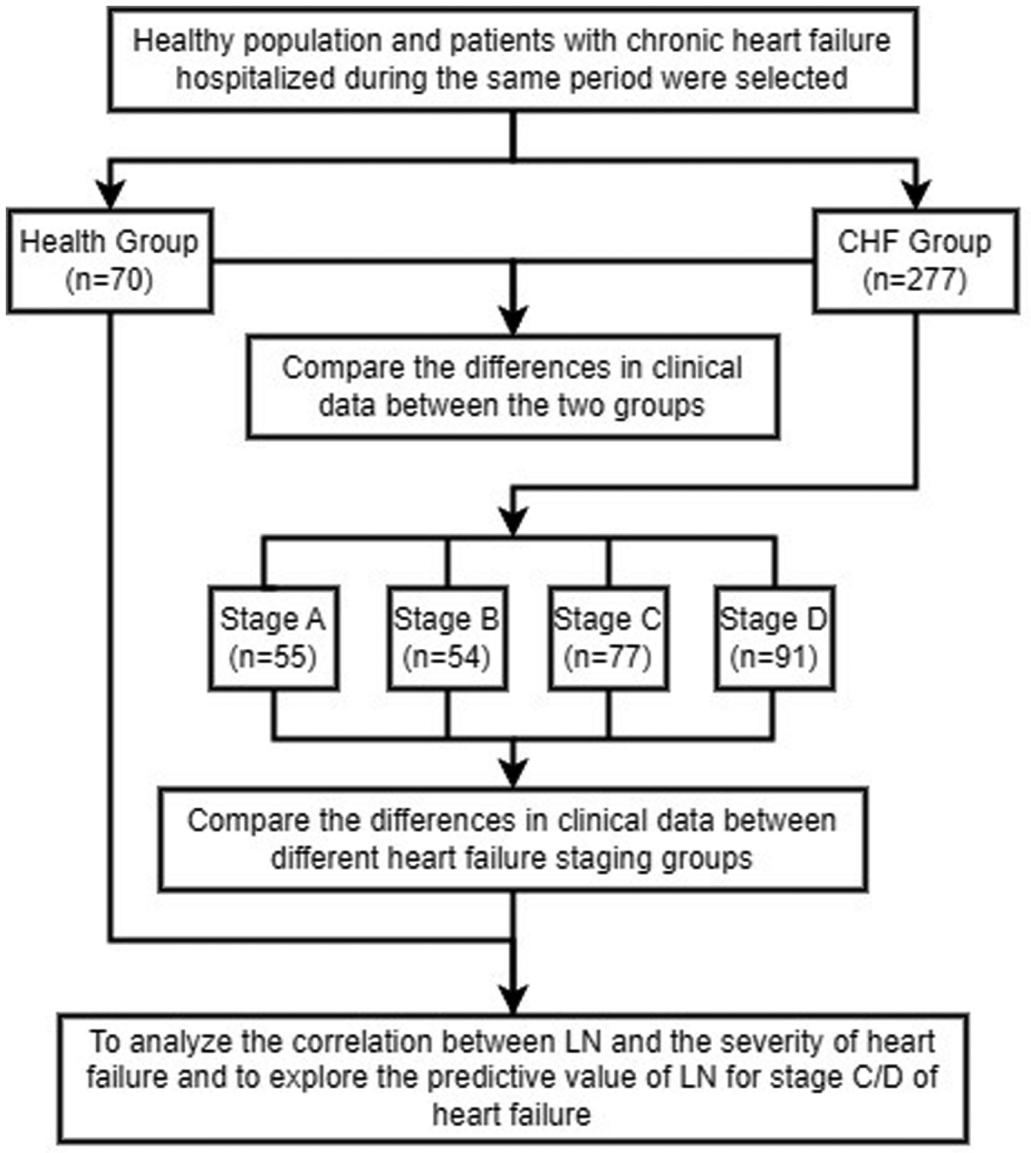
Overall technical roadmap.

We conducted an observational study from September 2019 to June 2020. 277 patients diagnosed as CHF in the Department of Cardiovascular Medicine of the Second Affiliated Hospital of Nantong University were selected. All patients met the diagnostic criteria of CHF, as shown in Figure 1 (8). Patients with rheumatic system diseases, liver fibrosis, malignant tumor and patients with incomplete clinical data were excluded. 70 healthy individuals who had no major physical illnesses and no chronic diseases such as cardiac/metabolic diseases at the time of physical examination at our medical examination center during the same period were selected as the control group. We collected relevant medical records of the patients, including past history and liver and kidney function indexes, and analyzed serum levels of LN, HA, and NT-proBNP, and detected LVEF values by echocardiography for the study, which was investigated in accordance with the principles in the Declaration of Helsinki. The study was approved by the Ethics Committee of the Second Affiliated Hospital of Nantong University (IRB: No. 2020KN094), and informed consent was obtained from all patients.

#### 2.1.2. Data collection

Basic information of patients is recorded at admission, including gender, age, BMI, Medical history and family history, blood pressure and heart rate at admission, etc. cardiac troponin I (cTnI) were measured immediately after admission, and left ventricular ejection fraction (LVEF) was recorded by echocardiography. The levels of serum laminin (LN), hyaluronic acid (HA), N-terminal pro-brain natriuretic peptide (NT-proBNP) and alanine aminotransferase (ALT), aspartate aminotransferase (AST), total bilirubin (Tb), serum creatinine (Scr), urea nitrogen (UN), high-sensitivity C-reactive protein (CRP), Fasting glucose, Hemoglobin, Platelet, Triglycerides, Total cholesterol, high-density lipoprotein cholesterol (HDL-C), Low-density lipoprotein cholesterol (LDL-C) were detected on an empty stomach on the early morning of the second day of hospitalization.

#### 2.1.3. The definition of clinical degree of chronic heart failure

Stage A: Pre-heart failure stage; Stage B: Pre-clinical heart failure stage; Stage C: Clinical heart failure stage; Stage D: Refractory end-stage heart failure stage (8). (Cardiac function staging is independently evaluated by two physicians. Once there is any inconsistency, it will be discussed and decided by another chief physician) Patients in the C/D stage of HF have an impact on their daily life and are often considered to be in a relatively severe stage.

#### 2.1.4. Detection of data

On the second morning after admission, 4 ml fasting blood was taken from all patients to measure serum laminin. Fasting blood was centrifuged for 10 min at a rate of 2,500 r/min, and then serum laminin levels were measured by chemiluminescence immunoassay (MAGLUMI 2000). The test kit is provided by Fosun Diagnostics Company (Changsha, China). The determination of serum level of HA was performed with the same method. The normal reference range for the serum laminin kit in this study is 0.51–50 ng/ml, and the normal reference range for hyaluronic acid is 0–100 ng/ml. NT-pro BNP was measured using a chemiluminescence technique (on Cobas E. from Roche).

#### 2.1.5. Detection of LVEF

All subjects were examined with a Philips EPIQ 7C cardiovascular ultrasound S5–1 adult probe/frequency 1–5 MHz, performed and calibrated by an experienced sonographer. During the examination, patients were instructed to lie on the left side, breathe calmly, connect to the ECG, and acquire the left ventricular apical four-chamber and two-chamber views, respectively, and scan the left ventricular long-axis view and series of short-axis views, and calculate the left ventricular ejection fraction using the Simpson biplane method, which is a two-dimensional mode in which systolic and diastolic volumes are assessed in two different planes in the apical four-chamber and apical two-chamber hearts. The endocardial margin of the left ventricle, including the papillary muscle, was traced using the trackball tracing in both systole and diastole. LV ejection fraction = (LV end-diastolic volume − LV end-systolic volume)/LV end-diastolic volume × 100%.

#### 2.1.6. Statistical analysis

Continuous variables of normal distribution are expressed as mean values ± standard deviation, while variables conforming to skewed distribution are expressed as median (25th percentile–75th percentile). Statistics are expressed as percentages or frequencies. Independent sample t-test was used for the measurement data of normal distribution, *χ*^2^ test was used for the counting data, and the MannWhitney U test was used for non-normally distributed measures between two groups, and the Kruskal Wallis rank sum test was used for non-normally distributed measures between multiple groups. The correlation between LN and NT-proBNP and LVEF was studied by Spearman's Rank Coefficient of Correlation, the predictive value of LN in C-D stage of HF was evaluated by ROC curve, and the independent factors influencing the severity of HF were studied by Logistic multivariate ordered analysis. The data were analyzed by SPSS 26.0 (SPSS Inc., Chicago, IL), *P* < 0.05 was considered statistically significant.

## 3. Results

### 3.1. Comparison of baseline data between heart failure group and normal group

**Figure 2:**
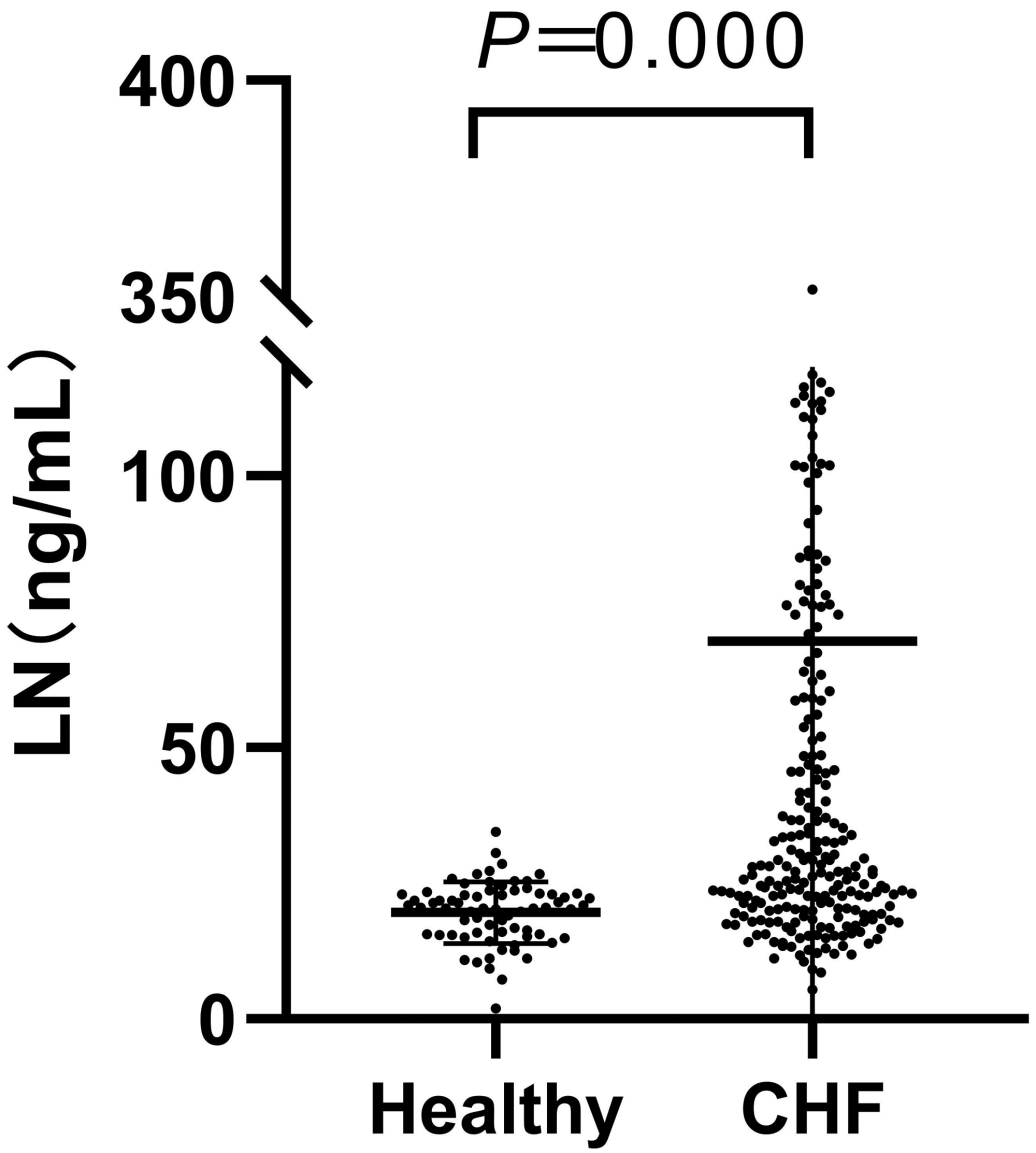
Serum laminin levels in healthy group and chronic heart failure group.

LN level in CHF group was significantly higher than that in healthy group, which were 33.2 (21.38, 101.9) ng/ml and 20.45 (15.53, 23.04) ng/ml respectively (*P *< 0.01, Figure 2). The data of NT-proBNP, HA, cTnI, Scr, Tb, AST, ALT, high-sensitivity CRP, fasting glucose, Systolic and male proportion of CHF group were significantly higher than those in healthy group, and LVEF value, Hemoglobin, HDL-C was lower than that in healthy group (*P *< 0.05). There was no significant difference in UN, age and sex (*P* > 0.05), as shown in Table 1.

**Table 1:** Comparison of general clinical information between the Two groups.

	Healthy group (*n* = 70)	CHF group (*n* = 277)	*P*
Male, *n* (%)	40, 57.1	189, 68.2	.080
Age, years	63 (55.75, 72.25)	68 (57, 75)	.095
BMI, kg/m^2^	24.79 (22.01, 27.32)	24.77 (23.4, 26.98)	.448
Systolic pressure (mmHg)	137 (124.5, 158)	127 (113, 139.75)	.000*
Diastolic pressure (mmHg)	79 (70.5, 86)	76.5 (68, 86)	.411
Heart rate (bpm)	80 (70, 90)	80 (70, 90)	.838
Smokers (*n*, %)	13, 18.6	57, 20.6	.709
Alcohol intake (*n*, %)	16, 22.9	43, 15.5	.144
Exercise (*n*, %)	26, 37.1	99, 36.7	.827
**Laboratory data**
Hemoglobin (g/L)	140 (132, 149)	134 (121, 149)	.045*
Platelet (×10^9^/L)	203 (163, 227)	185 (154, 228.5)	.403
Total bilirubin (µmol/L)	12.75 (9.48, 16.43)	14.45 (10.48, 20.53)	.027
Serum creatinine (µmol/L)	59.25 (50.95, 71.8)	71 (57.78, 89.03)	.000*
Urea nitrogen (mmol/L)	5.76 (4.76, 6.85)	5.74 (4.63, 7.73)	.648
ALT (U/L)	17 (11, 28.25)	25 (16.75, 39)	.000*
AST (U/L)	18.5 (16, 23)	26 (19, 55.75)	.000*
FBG (mmol/L)	5.14 (4.74, 5.83)	5.56 (4.89, 6.43)	.019*
High-sensitivity CRP (mg/L)	0.63 (0.1, 2.48)	3.09 (0.55, 8.78)	.000*
Triglycerides (mmol/L)	1.33 (0.96, 1.87)	1.18 (0.79, 1.67)	.060
Total cholesterol (mmol/L)	3.97 (3.50, 4.62)	3.98 (3.36, 4.54)	.243
HDL-C (mmol/L)	1.24 (1.08, 1.56)	1.08 (0.89, 1.34)	.000*
LDL-C (mmol/L)	2.47 (1.97, 2.98)	2.44 (1.87, 3.01)	.394
cTnI (µg/L)	0.01 (0.01, 0.01)	0.03 (0.01, 1.38)	.000*
NT- proBNP (pg/ml)	42.55 (20.33, 110.73)	1,383.7 (91.3, 4,626)	.000*
LN (ng/ml)	20.45 (15.53, 23.04)	33.2 (21.38, 101.9)	.000*
HA (ng/ml)	55.47 (50.69, 65.15)	76.23 (60.79, 103.45)	.000*
LVEF (%)	66 (63.75, 69)	60 (45, 66)	.000*
**Comorbidities**
Hypertension (*n*, %)	0, 0	143, 51.6	.000*
Diabetes (*n*, %)	0, 0	66, 23.8	.000*
Anemias (*n*, %)	0, 0	65, 23.5	.000*
Stroke (*n*, %)	0, 0	22, 7.9	.015*
Asthma (*n*, %)	0, 0	7, 2.5	.179
COPD (*n*, %)	0, 0	16, 5.8	.040*
Carotid artery disease (*n*, %)	0, 0	88, 25.4	.000*
Atrial fibrillation (*n*, %)	0, 0	80, 28.9	.000*
Valve disorder (*n*, %)	0, 0	67, 24.2	.000*
Heart failure (*n*, %)	0, 0	277, 100	.000*
Metabolic syndrome (*n*, %)	0, 0	10, 3.6	.107
Hypothyroidism (*n*, %)	0, 0	26, 9.4	.008*
Gout (*n*, %)	0, 0	49, 17.7	.000*
Liver steatosis (*n*, %)	0, 0	14, 5.1	.055
Chronic kidney disease (*n*, %)	0, 0	42, 15.2	.001*
Dialysis (*n*, %)	0, 0	5, 1.4	.258
GIB history (*n*, %)	0, 0	11, 4	.090
Drug abuse (*n*, %)	0, 0	8, 2.9	.150
Mental disorders (*n*, %)	0, 0	9, 3.2	.127
Depression (*n*, %)	0, 0	6, 1.7	.214
**Family medical history**
Family history of stroke (*n*, %)	7, 10	22, 7.9	.578
Family history of CAD (*n*, %)	5, 7.1	56, 20.2	.450
Family history of PAD (*n*, %)	3, 4.3	33, 11.9	.553

Values are reported as the number (%) or median (25th and 75th percentiles). ALT, alanine aminotransferase; AST, aspartate aminotransferase; FBG, fasting glucose; CRP, C-reactive protein; HDL-C, high-density lipoprotein cholesterol and LDL-C Low-density lipoprotein cholesterol; cTnI, cardiac troponin I; NT-proBNP, N-terminal pro-brain natriuretic peptide; LN, Laminin; HA, hyaluronic acid; LVEF, left ventricular ejection fraction; GIB, Gastrointestinal bleeding; CAD, Carotid artery disease; PAD, Peripheral arterial disease.

Values with *P* < 0.05 in the table are marked with an asterisk.

### 3.2. Baseline data comparison of four groups of patients with heart failure in different clinical stages

**Figure 3:**
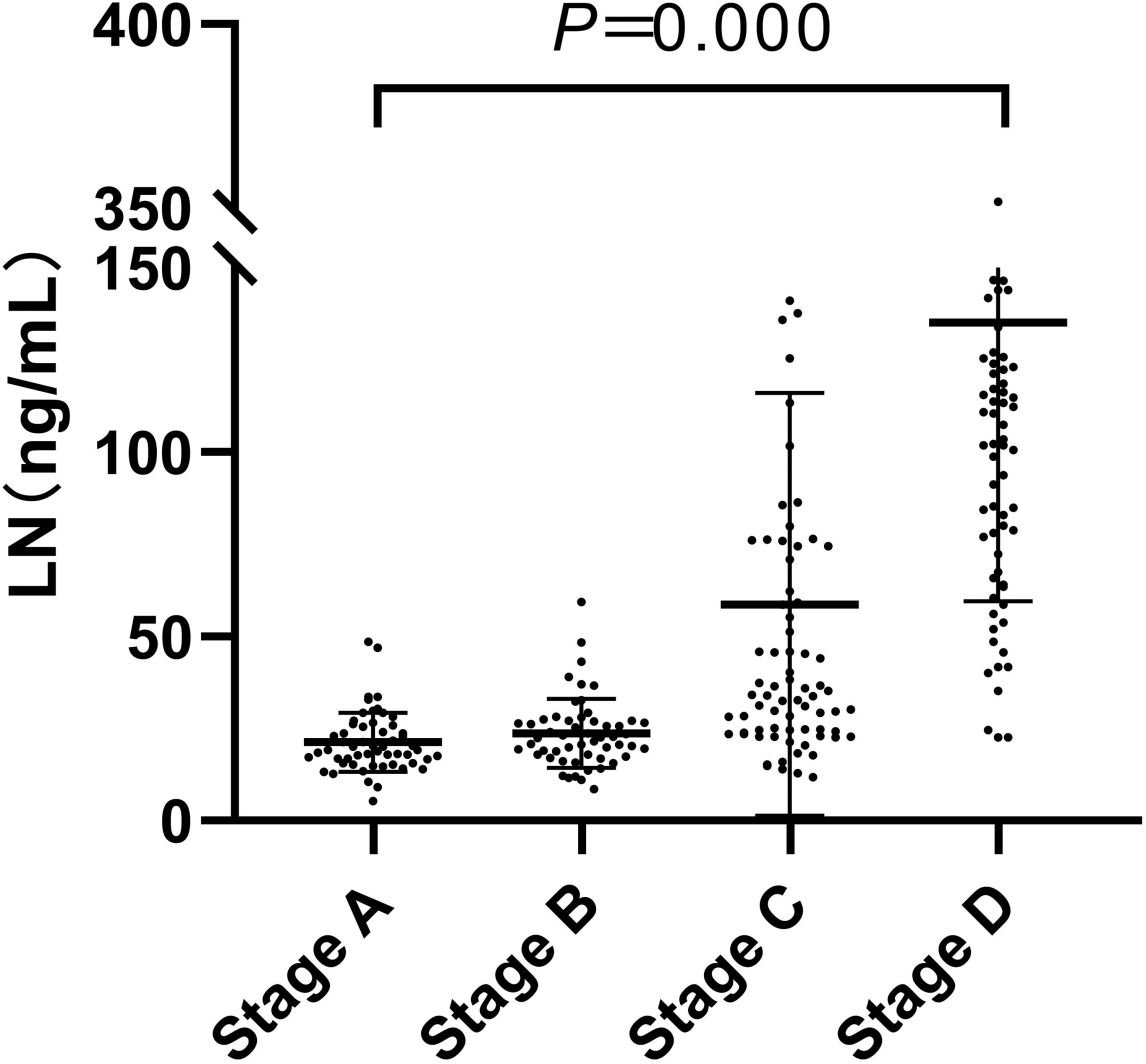
Serum laminin levels in patients with different stages of heart failure.

LN gradually increased with the progress of HF stages (*P* < 0.01, Figure 3), age, ALT, AST, Tb, Scr, UN, TnI, NT-proBNP, HA also gradually increased with the increase of clinical stages, LVEF value tended to decrease with the progress of heart failure stages (*P* < 0.01), and there was no statistical difference in the history of type 2 diabetes, hypertension and gender (*P* > 0.01), as shown in Table 2.

**Table 2:** Characteristics of patients with different stages of heart failure.

	Total (*n* = 277)	Stage A (*n* = 55)	Stage B (*n* = 54)	Stage C (*n* = 77)	Stage D (*n* = 91)	*P*
Male, *n* (%)	189, 68.2	31, 56.4	36, 66.7	62, 80.5	60, 65.9	.027*
Age, years	68 (57, 75)	62 (54, 70)	66.5 (57, 72.25)	67 (54.5, 76)	71 (65, 76)	.000*
Hypertension, *n* (%)	143, 51.6	26, 47.3	35, 64.8	40, 51.9	42, 46.2	.153
Diabetes, *n* (%)	66, 23.8	8, 14.5	16, 29.6	22, 28.6	20, 22	.192
ALT (U/L)	25 (16.75, 39)	23 (15, 30)	22 (16, 31.5)	34 (21, 53)	24 (13, 39.75)	.000*
AST (U/L)	26 (19, 55.75)	19 (16, 23)	20 (16.75, 27)	81 (34.5, 177.5)	30 (21, 43.25)	.000*
Total bilirubin (µmol/L)	14.45 (10.48, 20.53)	11.6 (9.7, 15.9)	11.7 (8.02, 15.13)	16 (10.95, 20.65)	19.55 (13.4, 29.8)	.000*
Scr (µmol/L)	71 (57.78, 89.03)	58.5 (53, 74)	65.05 (57.7, 75.15)	70 (55.8, 83.9)	91.65 (74.25, 120.05)	.000*
Urea nitrogen (mmol/L)	5.74 (4.63, 7.73)	5.5 (4.48, 6.22)	5.33 (4.19, 6.39)	5.08 (4.1, 7.05)	7.89 (6.1, 10.6)	.000*
cTnI (µg/L)	0.03 (0.01, 1.38)	0.01 (0.01, 0.01)	0.01 (0.01, 0.01)	9.96 (1.63, 42.75)	0.03 (0.02, 0.98)	.000*
NT-proBNP (pg/ml)	1,383.7 (91.3, 4,626)	85.9 (35.2, 126.6)	63.45 (19.15, 114.53)	1,990 (1,230.4, 3,960.5)	6,272 (3,955, 15,388)	.000*
LN (ng/ml)	33.2 (21.38, 101.9)	19.15 (15.51, 25.87)	22.55 (17.76, 27.1)	35.16 (24.37, 75.18)	117.9 (80.78, 172.7)	.000*
HA (ng/ml)	76.23 (60.79, 103.45)	58.67 (45.48, 70.05)	69.47 (60.7, 84.67)	75.88 (60.39, 100.81)	104 (81.3, 151)	.000*
LVEF (%)	60 (45, 66)	67 (64, 70)	65(60.75, 68)	56(49, 65)	38(30.25, 54.5)	.000*

Values are reported as the number (%) or median (25th and 75th percentiles). ALT indicates alanine aminotransferase; AST, aspartate aminotransferase; Scr, Serum creatinine; cTnI, cardiac troponin I; NT-proBNP, N-terminal pro-brain natriuretic peptide; LN, Laminin; HA, hyaluronic acid and LVEF, left ventricular ejection fraction.

Values with *P* < 0.05 in the table are marked with an asterisk.

### 3.3. The correlation analysis

**Figure 4:**
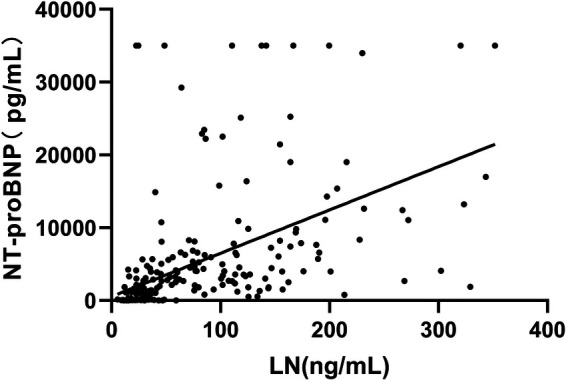
Correlation between LN and NT-proBNP.

**Figure 5:**
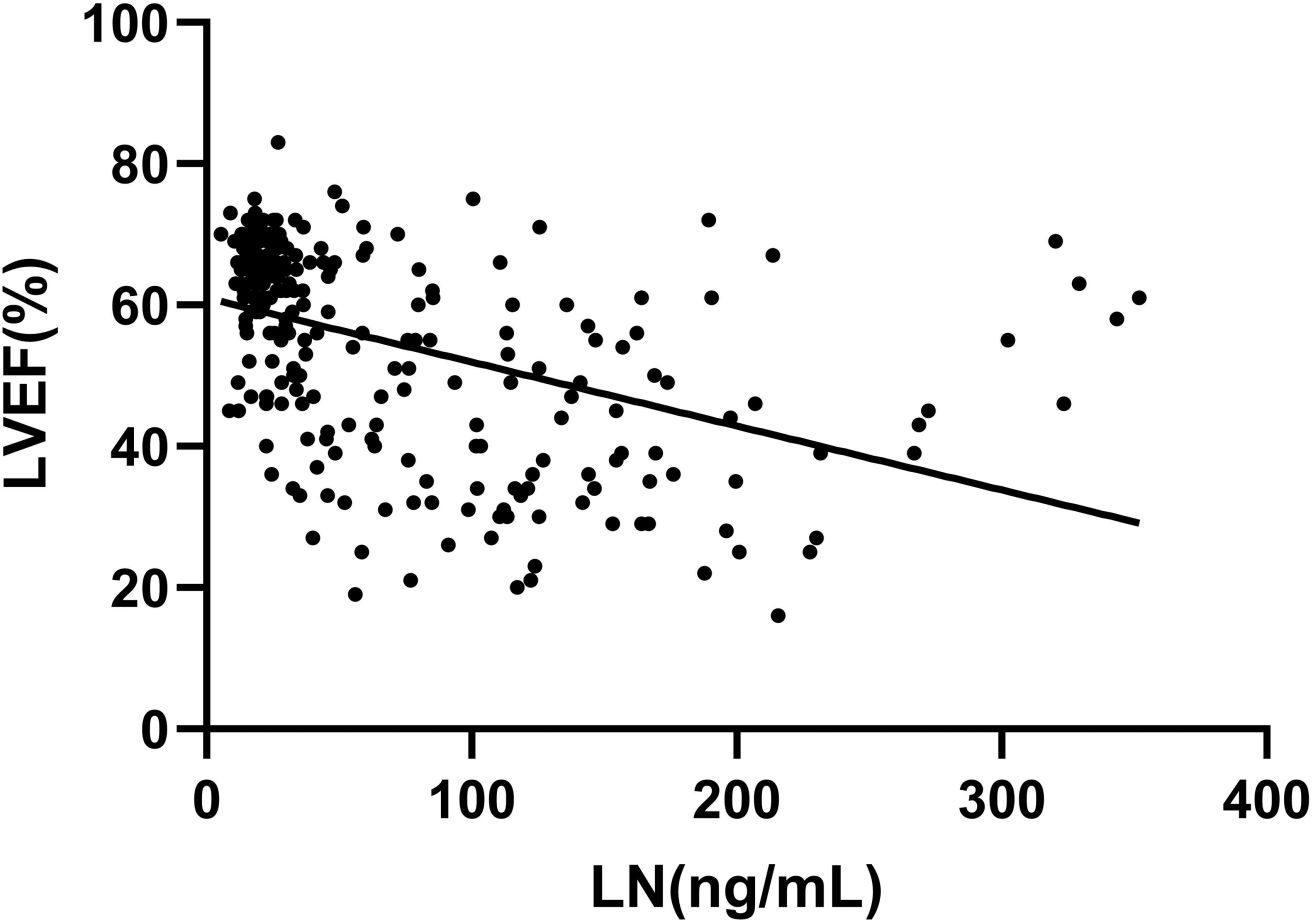
Correlation between LN and LVEF.

The correlation analysis between LN and NT-proBNP and LVEF showed that LN was positively correlated with NT-proBNP (*r* = 0.744, *P *= 0.000, Figure 4) while negatively correlated with LVEF (*r* = −0.568, *P *= 0.000, Figure 5).

### 3.4. ROC curve analysis

**Figure 6:**
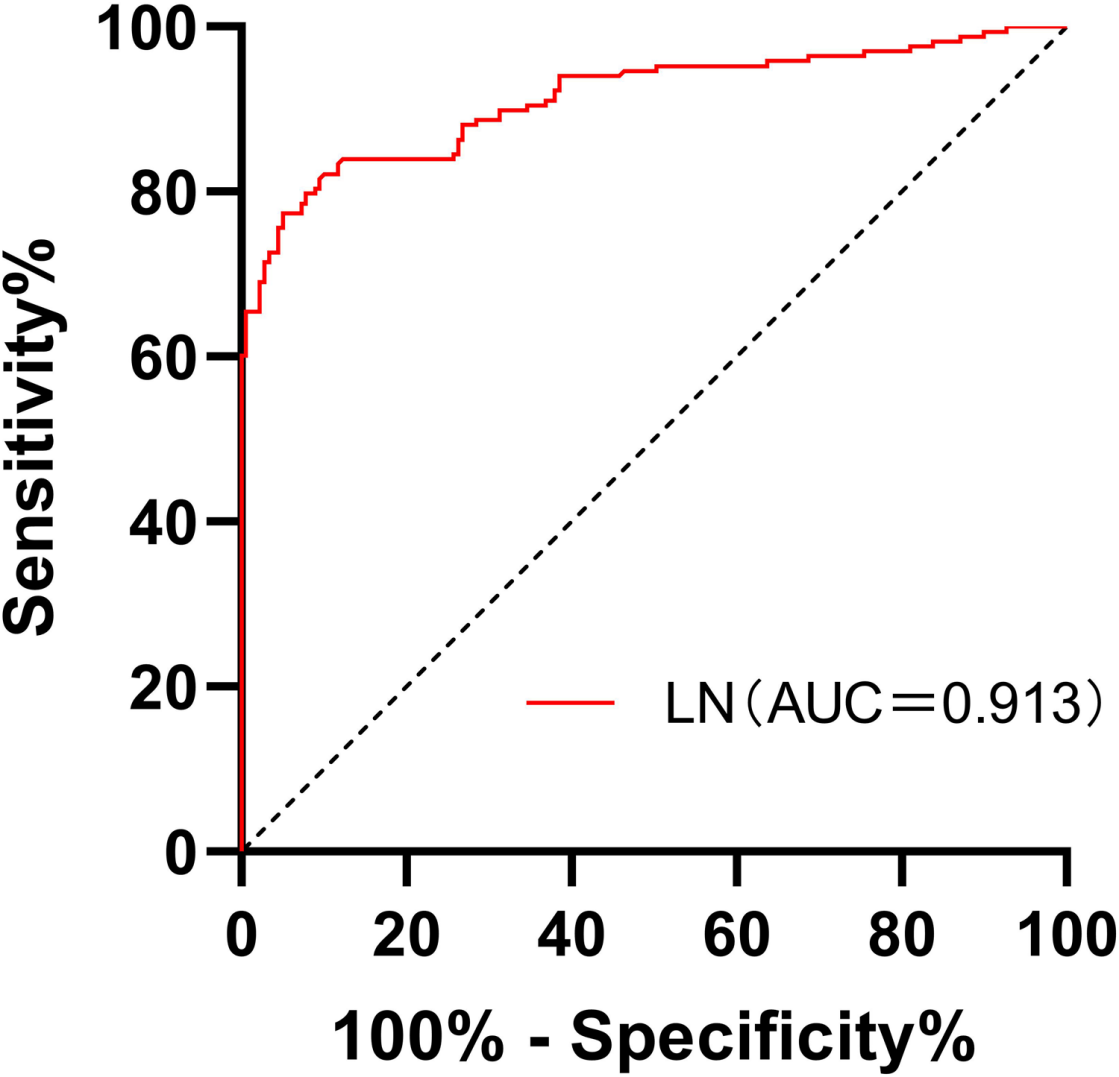
Receiver operating characteristic curves for laminin in the prediction of stages of C and D in CHF patients.

A total of 168 patients were enrolled in C-D stage of HF. The area of HF during C-D stage under ROC curve was predicted according to LN, which was 0.913, 95% confidence interval was 0.882–0.945, *P *= 0.000, cut-off value was 33.72 ng/ml, specificity 94.97% and sensitivity 77.38%. as shown in Figure 6 and Table 4

### 3.5. Predicting clinical outcome

Multivariate ordered Logistic analysis to different clinical stages of heart failure showed that LN (OR: 1.021, 95% CI: 1.011–1.030, *P *= 0.000), HA (OR: 1.011, 95% CI: 1.003–1.019, *P *= 0.005), NT-proBNP (OR: 1.000, 95% CI: 1.000–1.000, *P *= 0.017), LVEF (OR: 0.893, 95% CI: 0.865–0.923, *P *= 0.000), age (OR: 1.033, 95% CI: 1.006–1.061, *P *= 0.016), AST (OR: 1.006, 95% CI: 1.000–1.012, *P *= 0.048), and Tb (OR: 1.061, 95% CI: 1.027–1.097, *P *= 0.000) are all independent risk factors of severity of heart failure (*P* < 0.05), as shown in Table 3.

**Table 3:** Multivariate ordered logistic analysis to different clinical stages of heart failure.

	Unadjusted OR (95% CI)	*P*-value	Adjusted OR (95% CI)	*P*-value
Age, years	1.042 (1.022, 1.062)	0.000	1.033 (1.006, 1.061)	0.016
ALT (U/L)	1.012 (1.002, 1.021)	0.011		
AST (U/L)	1.003 (1.001, 1.006)	0.017	1.006 (1.000, 1.012)	0.048
Total bilirubin (µmol/L)	1.084 (1.055, 1.114)	0.000	1.061 (1.027, 1.097)	0.000
Serum creatinine (µmol/L)	1.023 (1.015, 1.032)	0.000		
Urea nitrogen (mmol/L)	1.274 (1.168, 1.389)	0.000		
cTnI (µg/L)	1.007 (0.996, 1.018)	0.223		
NT-proBNP (pg/ml)	1.000 (1.000, 1.001)	0.000	1.000 (1.000, 1.000)	0.017
LN (ng/ml)	1.038 (1.030, 1.046)	0.000	1.021 (1.011, 1.030)	0.000
HA (ng/ml)	1.0219 (1.015, 1.029)	0.000	1.011 (1.003, 1.019)	0.005
LVEF (%)	0.867 (0.845, 0.889)	0.000	0.893 (0.865, 0.923)	0.000

ALT indicates alanine aminotransferase; AST, aspartate aminotransferase; cTnI, cardiac troponin I; NT-proBNP, N-terminal pro-brain natriuretic peptide; LN, Laminin; HA, hyaluronic acid and LVEF, left ventricular ejection fraction.

Values with *P* < 0.05 in the table are shown in red.

**Table 4:** Receiver operating characteristic curves for laminin in the prediction of stages of C and D in CHF patients.

ROC curve statistics	**Sen**	**Spe**	**AUC**
	77.38%	94.97%	0.913
	**+PV**	**−PV**	**DOR**
	93.5	81.7	64.125
	**+LR**	**−LR**	**Youden index**
	15.39	0.24	0.724

Sen, sensitivity; Spe, specificity; +PV, positive predictive values; −PV, negative predictive values; +LR, positive likelihood ratios; −LR, negative likelihood ratios; AUC, area under the ROC curve; DOR, diagnostic odds ratio.

## 4. Discussion

HF (9) is a clinical syndrome with symptoms and signs caused by heart structure and/or function anomaly. It will ultimately lead to all chronic cardiovascular diseases and is one of the most harmful diseases to human health. During CHF progression, the whole myocardium undergoes extensive irreversible remodeling, and ventricular remodeling mainly includes myocardial cell injury and myocardial fibrosis (10, 11). In the past, researchers mainly focused on the study in the field of myocardial cell injury. At present, endomyocardial biopsy is used to diagnose myocardial fibrosis clinically, but it is difficult to carry out routinely due to the difficulties in obtaining materials and patients' acceptance (12). Therefore, biomarkers of myocardial fibrosis are widely accepted as alternative reference indicators clinically.

Myocardial fibrosis features by changes in ECM. Previous studies on ECM and myocardial fibrosis mainly focused on collagen types I and collagen type III, which were used as non-invasive monitoring methods for tissue and organ fibrosis (6, 13). Serum LN is an important part of ECM, which was first discovered in mouse tumor basement membrane by Timple and other researchers in 1979 (14), serum LN is one of the largest non-collagenous glycoproteins in the basement membrane and plays a key role in fibrosis formation. Studies have shown that various defective regenerative processes/unresolved inflammation and/or chronic injury endpoints include varying degrees of fibrosis (15). serum LN has been used to evaluate liver and pulmonary fibrosis in the past (16, 17).

Serum LN chain was confirmed to exist in the basement membrane area of the human embryo and fetal heart system by Roediger et al. (18) in 2010, and was proven to play a role in the development of the human circulatory system. Samura et al. found that serum LN is the most likely expressed cardiac LN, which can promote the differentiation of pluripotent human embryonic stem cells into myocardial cell lineage and promote cardiovascular progenitor cells to generate infarct human cardiac muscle *in vivo* (19). In 2016, Evelyn et al. (20) also discovered that serum LN participated in the periodic remodeling of cardiac basement membrane in rats with ischemic HF. HF could elevate the pressure in the central and peripheral veins, leading to congestion of passive organs in the abdominal cavity (21). The increase in sympathetic nerve activity further promoted the fibrosis process of the heart, kidney, and liver (22–24). Throughout these studies, it is evident that LN plays an important role in the fibrotic process in several organs, but no clinical studies have yet addressed the relevance of LN to the clinical staging of patients with CHF.

In recent decades, the prognosis of patients with HF has been greatly improved after standardized treatment. However, owing to the irreversibility of ventricular remodeling, the overall life quality still decreased significantly (25). The prognosis of patients with stages A-B of HF can be greatly improved with early intervention. Thus, this study aimed to explore the clinical significance of the changes in serum LN concentration in the during the development of different stages of CHF.

This study found that the serum LN level in patients with CHF was significantly higher than that in healthy people, and with the progress of clinical stages of HF, the serum LN level showed an increasing trend (*P* ≤ 0.001). LN was positively correlated with NT-proBNP (*r* = 0.744, *P *= 0.000) and negatively correlated with LVEF (*r* = −0.568, *P *= 0.000). The area under the ROC curve of LN for predicting C and D stages of HF was 0.913, 95% confidence interval was 0.882–0.945, *P *= 0.000, cut-off value was 33.72 ng/ml, specificity was 94.97%, and sensitivity was 77.38%. Multivariate logistic analysis proved that LN was an independent related factor in the stage of HF. Therefore, it is reasonable to speculate that serum LN is closely related to the process of myocardial fibrosis in CHF, and the concentration of serum LN could gradually increase with HF progression and continuous ventricular remodeling. Hawkes et al. (26) discovered that LN is abundant in the healthy heart, but fibronectin is increasingly expressed in the fibrotic heart. The traditional NT-proBNP is the most widely applied biomarker in the diagnosis and treatment of HF, which is of great significance in the process of diagnosis, treatment and prognosis evaluation of HF (8). However, NT-proBNP has been found to be higher or lower than the expected value of the disease state in clinics in recent years. It is susceptible to renal insufficiency, age, sex, atrial fibrillation and other factors, and could even show an abnormal “normalization” trend at the end stage of HF (27). Thus, the combination with LN, a fibrosis index, could more accurately evaluate the severity and clinical stage of HF when NT-proBNP is defective.

### 4.1. Limitations

This study has some limitations. This study lacks data on the severity of fibrosis, and as a retrospective study, it lacks dynamic observation of LN levels at multiple time points, which is worthy of further large-scale clinical and basic research to verify its conclusions.

## 5. Conclusion

In summary, the serum LN level in patients with CHF is obviously increased, which is independently related to the clinical stage of HF, and could potentially become an early warning index of the severity of the HF process.

## Data Availability

The original contributions presented in the study are included in the article/**Supplementary Materials**, further inquiries can be directed to the corresponding author.
