# Supplementary figures and images for: Correlations between serum laminin level and severity of heart failure in patients with chronic heart failure
Source: Front Cardiovasc Med. 2023 Mar 16;10:1089304. doi: 10.3389/fcvm.2023.1089304 (PMC10060624; doi:10.3389/fcvm.2023.1089304)

Supplementary Material

# Supplementary Data


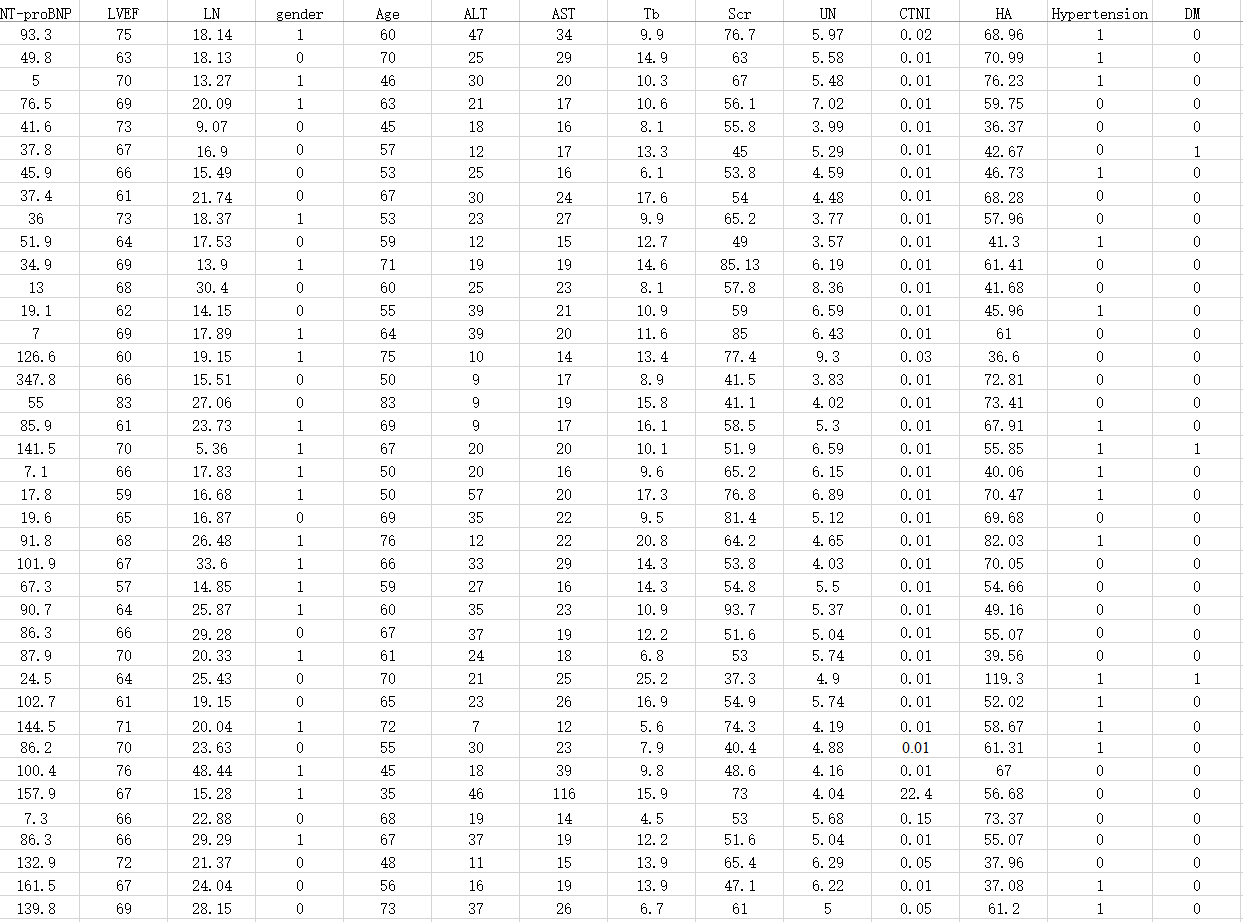


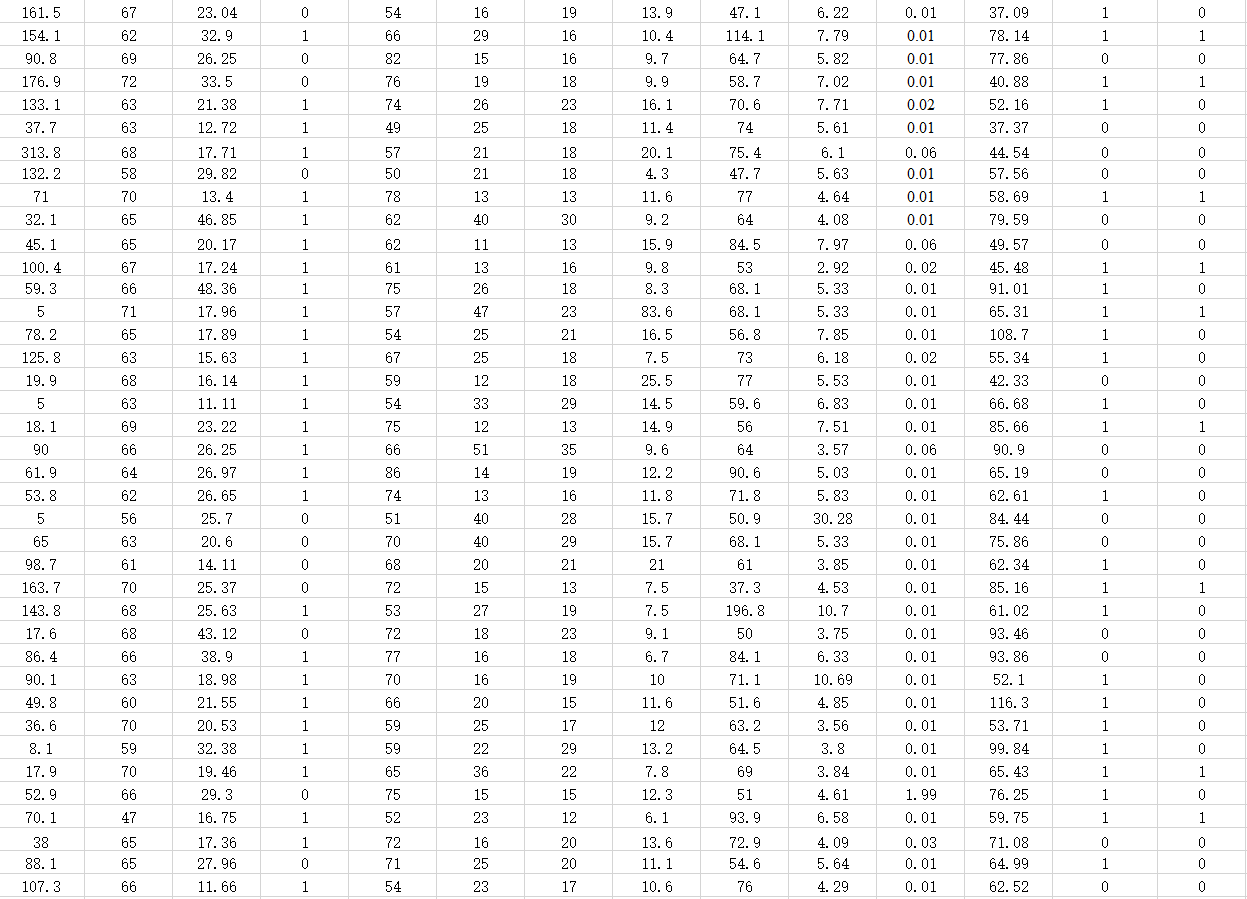


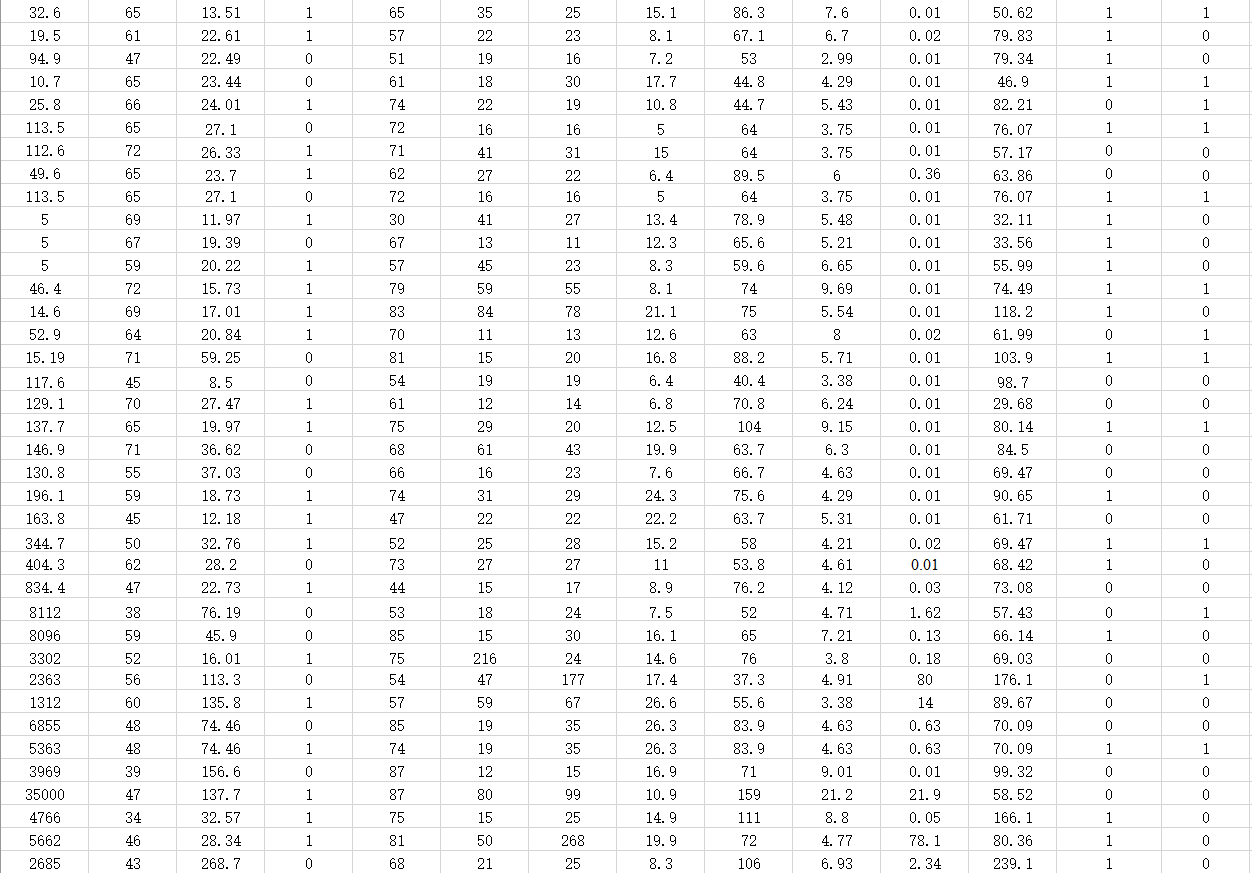


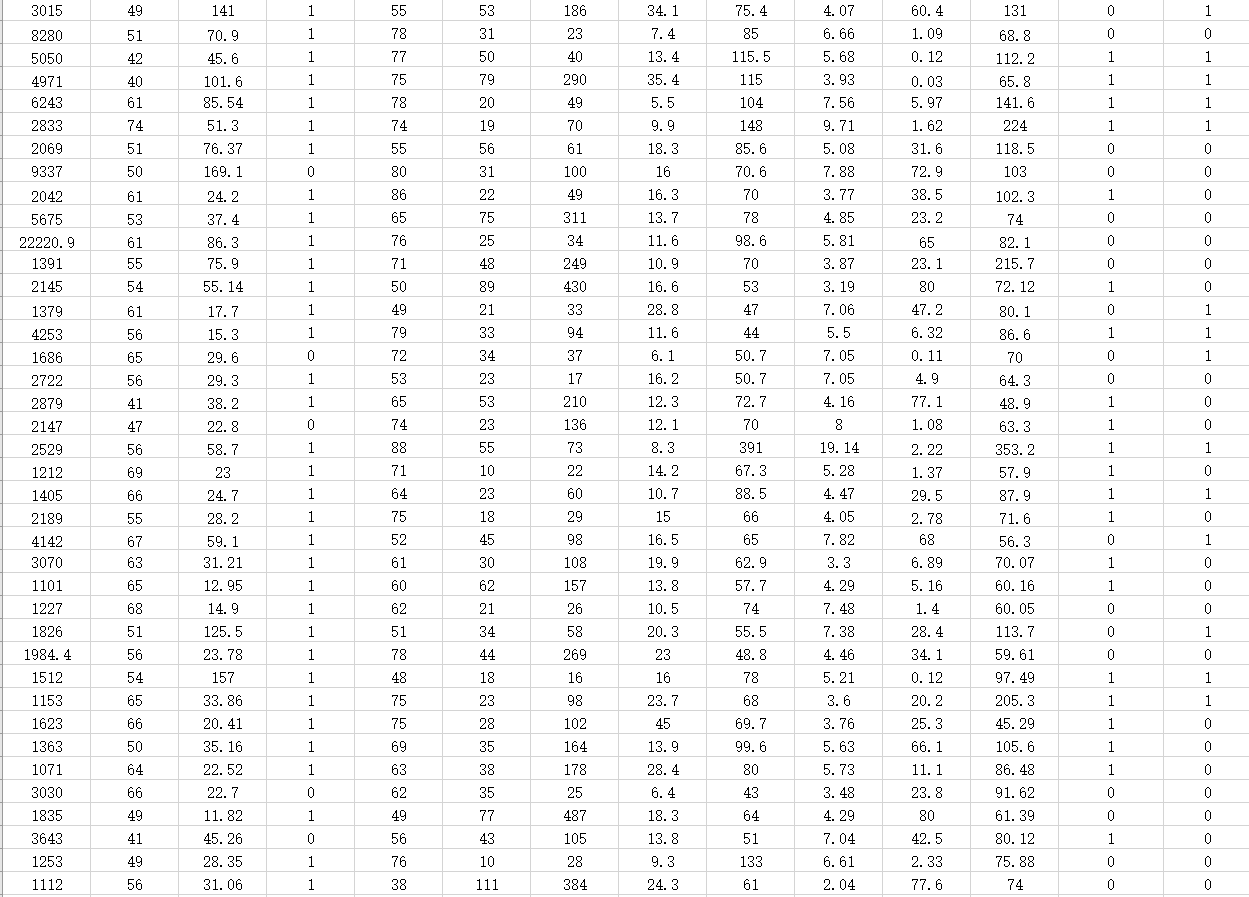


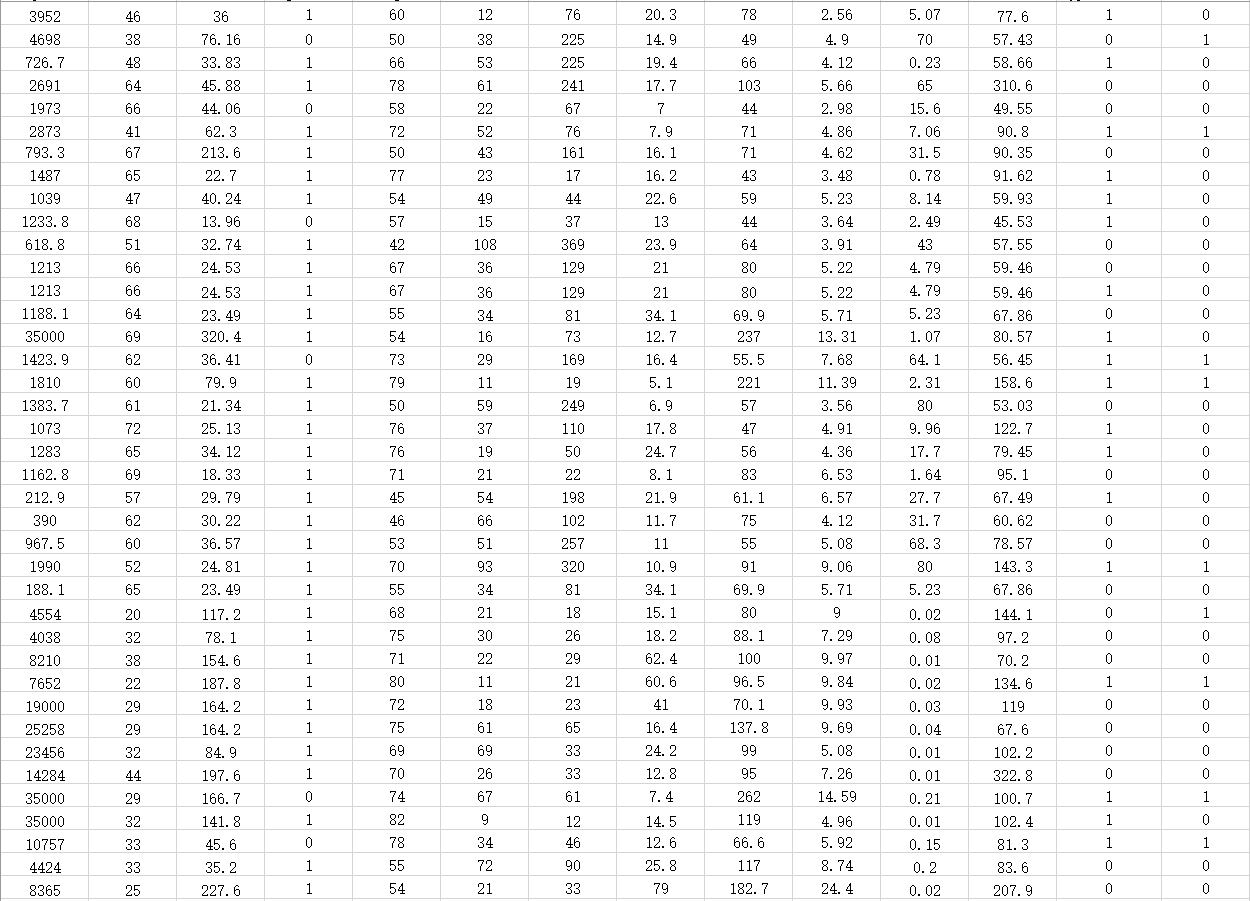


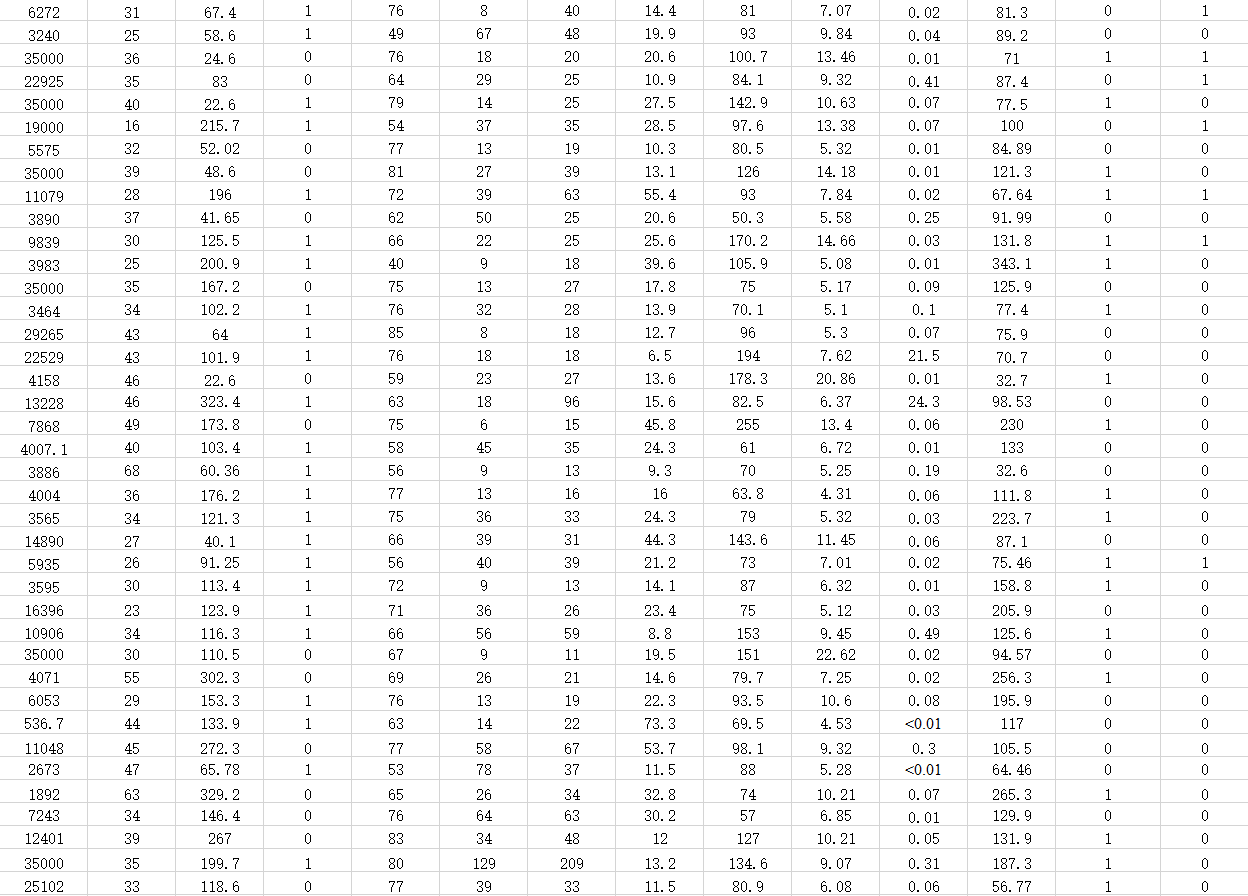


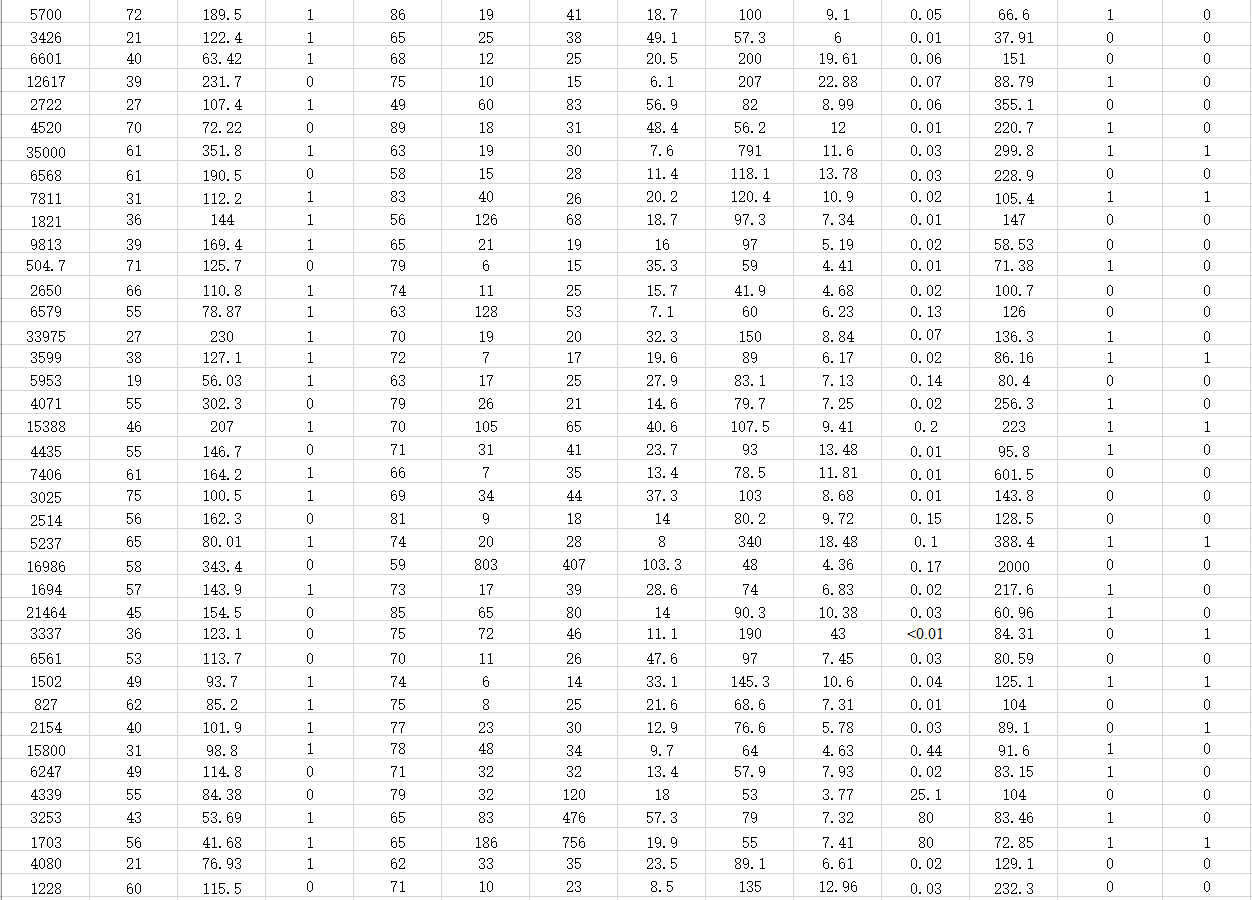


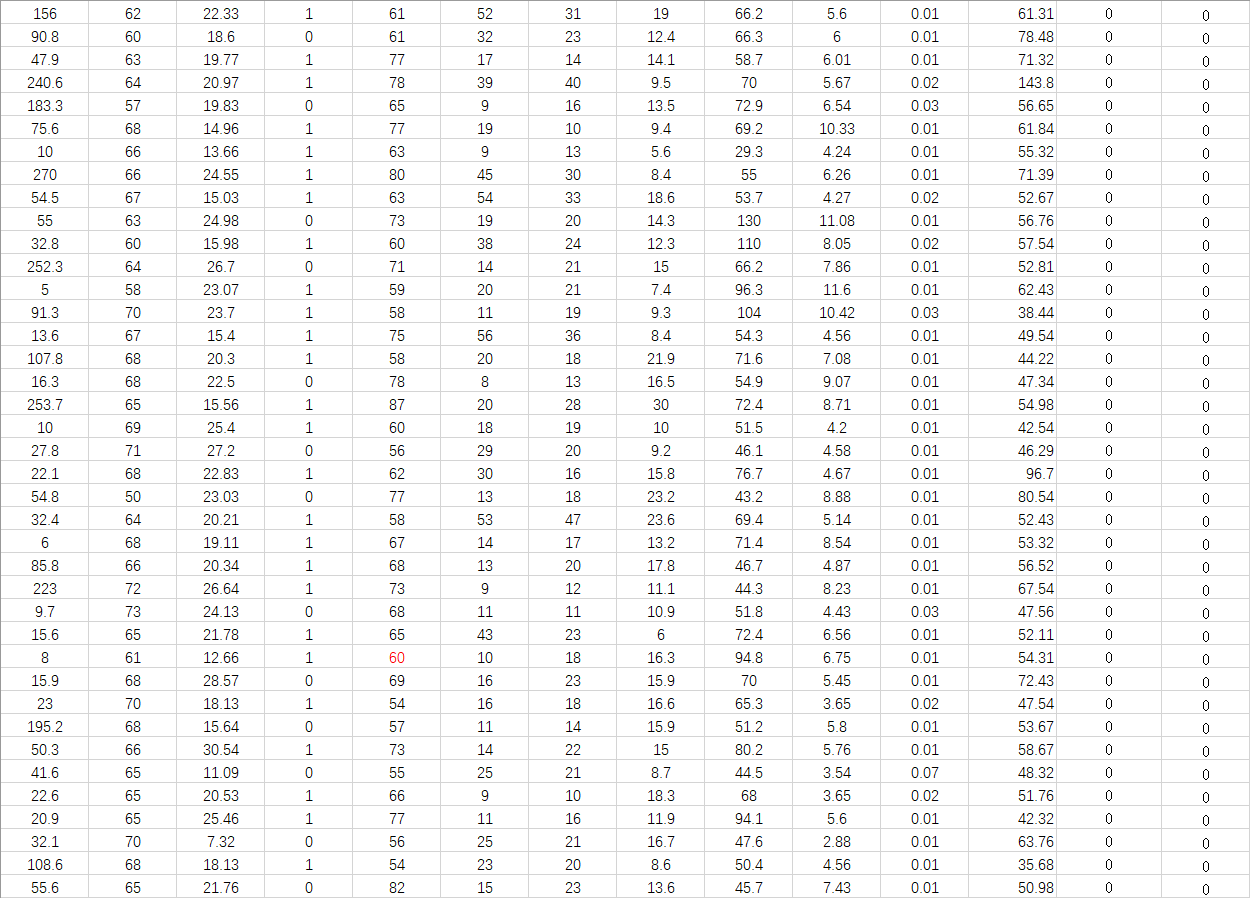

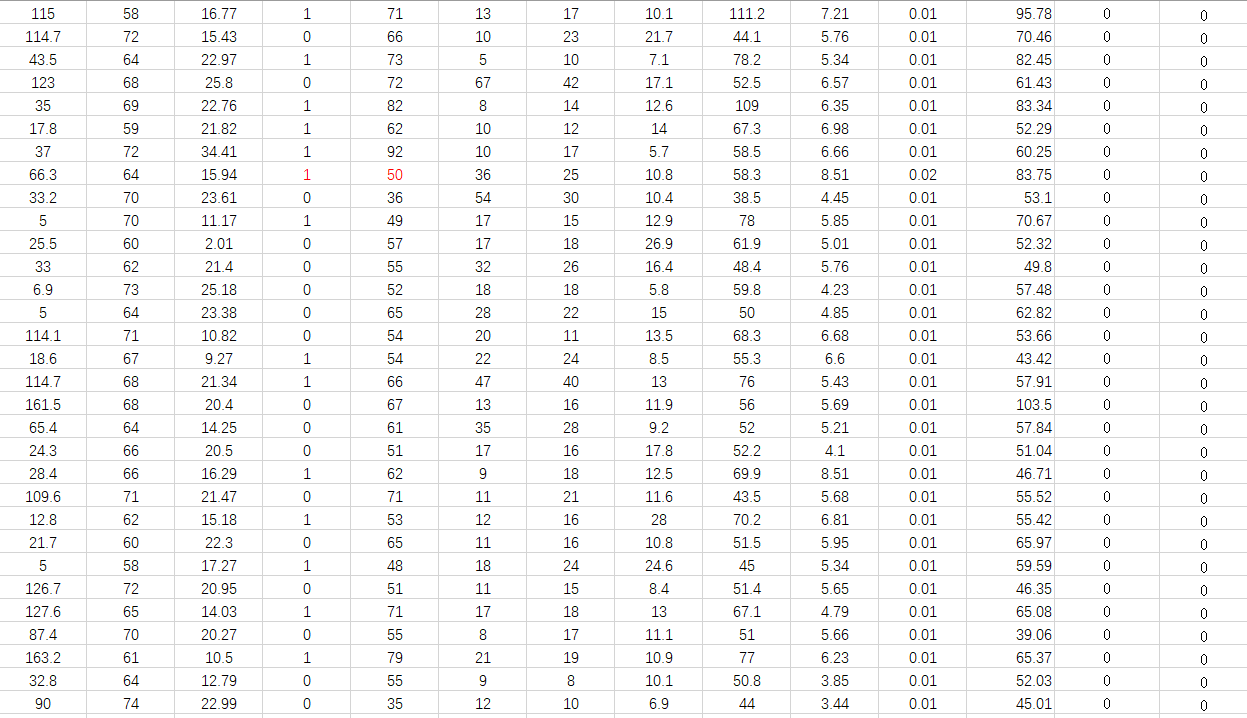

Supplement: Supplementary file 1 [file Table1.docx]
